# Giant Chondrosarcoma of Proximal Humerus in an Adult Female Patient: A Case Report

**DOI:** 10.5704/MOJ.1507.001

**Published:** 2015-07

**Authors:** CK Ng, A Azuhairy, LH Tan, A Nordin

**Affiliations:** Department of Orthopaedics, Seberang Jaya Hospital, Seberang Jaya, Malaysia; * Department of Orthopaedics, Pulau Pinang Hospital, Georgetown, Malaysia

**Keywords:** Giant chondrosarcoma, proximal humerus, young female, forequarter amputation

## Abstract

Chondrosarcoma is the third most common primary tumour of the bone, after myeloma and osteosarcoma. Most of the chondrosarcoma grow slowly and rarely metastasize, and they have an excellent prognosis after adequate surgery. However most of them are chemo or radio-resistant. We report a case of primary chondrosarcoma of proximal humerus in a 36-year-old female who presented with a six years history of left shoulder swelling and restricted range of motion. Trucut biopsy showed a well-differentiated chondrosarcoma. The patient underwent forequarter amputation of left upper limb and was started on chemotherapy following operation.

## Introduction

Primary bone tumours are uncommon and this has certainly contributed to the scarcity of data about their relative frequency, and to the limited understanding of the risk factors. Overall, bone sarcomas account for 0.2% of all malignancies, and the adjusted incidence rate for all bone and joint malignancies is 0.9 per 100,000 persons per year, while the 5-year overall survival rate is 67.9%^1^. Chondrosarcoma constitutes a heterogeneous group of neoplasms that have the production of cartilage matrix by tumour cells in common^2^. This primary sarcoma of bone in adults has a male predominance and is well demonstrated between the 3rd and 7th decades of life. Low grade chondrosarcoma has similar appearance to enchondroma and osteochondroma and has occasional binucleated cells. High grade chondrosarcoma has increased cellularity, atypia, and mitoses^3^. We report a case of a well-differentiated giant chondrosarcoma of the proximal left humerus in a 36-year-old female patient who underwent forequarter amputation with good recovery.

## Case Report

**Fig. 1:**
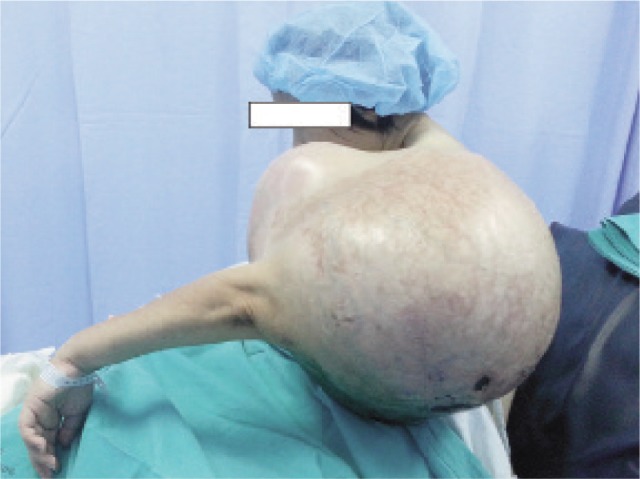
Giant chondrosarcoma of left proximal humerus.

A 36-year-old female patient was admitted to our department, presenting progressive enlarging mass and restricted range of motion of the left shoulder (Figure 1) for 6 years since year 2009. At the time of admission, she had completely lost occupational capacity of her left upper limb. Multiple foul smelling ulcers had developed over the swelling. Physical examination revealed a solid mass over proximal part of left upper limb measuring 450mm × 250mm with loss of normal contour of the left shoulder. Two discharging ulcers and a huge necrotic ulcer with slough were seen at the posterolateral and posterior surfaces respectively of the left arm. The swelling was bony hard in consistency but not tender on palpation. Dilated veins were noted on its surface. No lymph nodes were palpable over cervical and axillary regions. Computed tomography (CT) and Magnetic Resonance Imaging (MRI) were unable to be performed on admission due to its enormous size of swelling. Hence angiography of the left upper limb was performed to locate the anatomical position of major vessels and to detect anomalies as well. Trucut biopsy of the mass was performed and the histopathological examination showed fragments of tumour tissue composed of predominantly cartilaginous tissue with nuclei which were plump and hyperchromatic with occasional two nuclei per lacuna, which is consistent with well differentiated chondrosarcoma.

An informed consent was obtained from the patient prior to the operation. A multi-disciplinary intervention by the orthopaedic, radiological, anaesthetist and oncology team was adopted. She underwent forequarter amputation of her left upper limb. The tumour tissues were excised with maximum possible surgical safety margins. Intraoperatively there was neither excessive bleeding nor development of major complications. The resected mass measured 450 × 415 × 250mm and weighed 31kg (Figure 2a) and was submitted for histopathological examination. It was reported as a well differentiated chondrosarcoma. She was discharged uneventfully one month later with a well healed wound (Figure 2b). Prior to discharge CT thorax, abdomen and pelvis revealed no evidence of distant metastases. Adjuvant chemotherapy was initiated three months following the operation. The patient was advised for regular follow up to monitor the disease progression.

**Fig. 2a:**
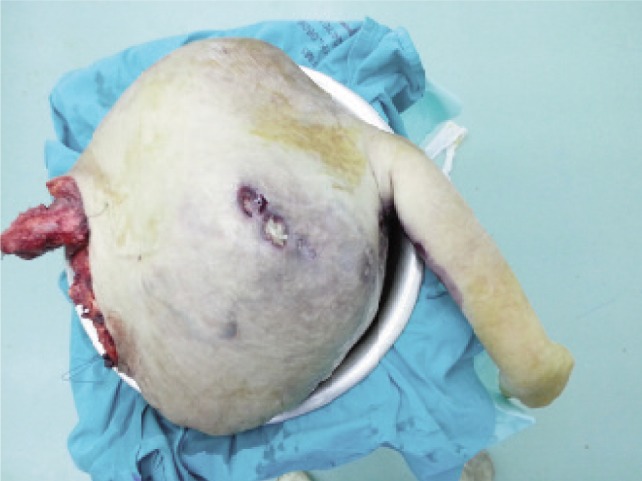
Excised tumour.

**Fig. 2b:**
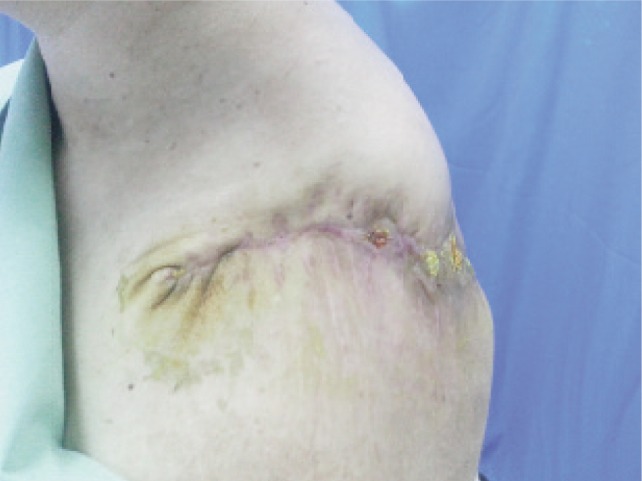
Four weeks post forequarter amputation.

### Histopathological findings (Figure 3)

Microscopically, the sections showed lobulated tumour composed of chondrocytes with hyperchromatic nuclei. There was minimal cellularity which exhibited rare small, dark nuclei and multinucleated forms. Mitotic activity was rare. Minimal tumour necrosis was present. Lymph vascular permeation -noted. There was no osteoid formation. The tumour cells involved part of the adjacent bone and its marrow, adjacent soft tissue and adjacent muscle. No joint or neurovascular bundle at margin were involved by tumour cells. Two lymph nodes were positive for metastatic chondrosarcoma.

**Fig. 3:**
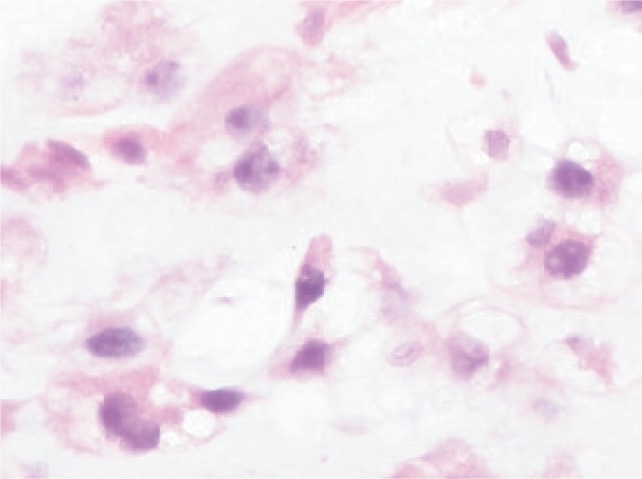
High power magnification of tumour showing malignant chondrocytes with hyperchromatic nuclei.

## Discussion

Chondrosarcoma of the proximal humerus is an uncommon malignant bone tumour, and limited information is available about treatment. Preservation of the functional capacities of the involved limb along with a complete removal of the tumour is considered as the most important criterion in the surgical management of tumour of the proximal humerus. Although literatures support wide local excision for Grade I conventional chondrosarcoma rather than amputation, decision of forequarter amputation was made after taking into consideration of the tumour size which was too extensive and it was fungating and ulcerated. Furthermore the neurovascular structures of left upper extremity were severely affected. Therefore forequarter amputation with adequate surgical margin remained the best option procedure.

Forequarter amputation of her left upper extremity posed challenges to the surgeon due to the enormous size of the tumour. Pre-operative planning with MRI and CT scan were not possible to be performed. It made the resection more challenging as we could not assess the extent of the chondrosarcoma and delineate the extent of soft tissue involvement for clear resection of the tumour. With the help of angiography of left upper limb the risk of bleeding during operation was minimized as anatomical position of major vessels were identified and no other anomalies such as vessel aneurysm was detected pre-operatively.

Chondrosarcoma of bone generally has a good prognosis when optimally diagnosed at an early stage. Histopathological grading, at present, is the best predictor of clinical behaviour. A series from the 1980s reported a 5-9% risk of metastasis with low grade conventional chondrosarcoma, whereas recent series reported 3% or no risk of metastasis^4^. The prognosis of this patient is good in view of the histopathological finding of the tumour which supports grade I chondrosarcoma. Although patient defaulted treatment for 6 years, there was no evidence of distant metastasis in solid organs based on CT scan done postoperatively.

It is generally believed that chondrosarcoma is relatively chemo- and radiotherapy resistant due to the extracellular matrix, poor vascularity and low percentage of dividing cells. However the patient was initiated on adjunct chemotherapy in view of her young age, high tumour load and evidence of regional lymph node- metastasis with adjacent bone marrow involvement.

Currently the patient is on six monthly follow up and she is satisfied with her current condition. She is looking forward to finding a new job after completing her chemotherapy.
